# Evolution of iris colour in relation to cavity nesting and parental care in passerine birds

**DOI:** 10.1098/rsbl.2016.0783

**Published:** 2017-01

**Authors:** Gabrielle L. Davidson, Alex Thornton, Nicola S. Clayton

**Affiliations:** 1 Department of Psychology, University of Cambridge, Cambridge CB2 3EB, UK; 2 School of Biological, Earth and Environmental Sciences, University College Cork, Cork, Ireland; 3 Centre for Ecology and Conservation, University of Exeter, Penryn Campus, Penryn TR10 9FE, UK

**Keywords:** colour evolution, iris colour, birds, nesting, parental care, passerines

## Abstract

Strong selection pressures are known to act on animal coloration. Although many animals vary in eye colour, virtually no research has investigated the functional significance of these colour traits. Passeriformes have a range of iris colours, making them an ideal system to investigate how and why iris colour has evolved. Using phylogenetic comparative methods, we tested the hypothesis that conspicuous iris colour in passerine birds evolved in response to (a) coordination of offspring care and (b) cavity nesting, two traits thought to be involved in intra-specific gaze sensitivity. We found that iris colour and cooperative offspring care by two or more individuals evolved independently, suggesting that bright eyes are not important for coordinating parental care through eye gaze. Furthermore, we found that evolution between iris colour and nesting behaviour did occur in a dependent manner, but contrary to predictions, transitions to coloured eyes were not more frequent in cavity nesters than non-cavity nesters. Instead, our results indicate that selection away from having bright eyes was much stronger in non-cavity nesters than cavity nesters, perhaps because conspicuous eye coloration in species not concealed within a cavity would be more visible to predators.

## 1. Introduction

Coloration is a prominent animal phenotype that is essential for several aspects of signalling including aposematism [1], species recognition [2] and sexual selection [3]. Despite the adaptive function of colour, scant attention has been paid to an important coloured phenotype—the eyes. In humans, laterally elongated eyes with a conspicuous white sclera around the iris are thought to have evolved specifically for cooperative communication through eye gaze (e.g. [4]). By contrast, other primates have round, dark eyes, which may be beneficial when concealing gaze from competitors and appearing less conspicuous to predators [4]. Many birds [5], amphibians [6] and fish [7] have conspicuous eyes, but the function of iris colour and its role in communication outside the primate lineage is poorly understood.

It has been proposed that eye coloration may be related to ecology [5,6], aggression (e.g. [7]), mate recognition and/or sexual selection (e.g. [6]). Passeriformes is the largest order of birds, and has a wide range of iris colour across species, making it an ideal system for studying the evolution and function of eye coloration. Two main hypotheses have been proposed as functions of iris coloration in birds. First, conspicuous eyes may be important for communicating to competitors—the white iris in jackdaws (*Corvus monedula*) may signal to conspecifics to keep away from occupied cavities [8]. Second, although yet to be tested empirically, conspicuous eyes may be important for within-pair communication, by highlighting salience of gaze direction [9]. Using eye gaze to coordinate actions such as building nests (e.g. indicating where to place nest material), provisioning (e.g. indicating which chick to feed) and nest guarding (e.g. spotting the location of predators) may strengthen bonds and improve reproductive success. Here, we test the hypothesis that bright irides as opposed to dark irides are more likely to evolve in cavity nesting birds than non-cavity nesting birds, and/or in birds where two or more individuals contribute to coordinated parental care rather than there being no coordinated parental care.

## 2. Material and methods

We generated a database of iris coloration, nesting behaviour and parental care for Passeriformes, as well as sister clades, Psittacidae and Falconiformes as outgroups [10]. Iris colour (bright/dark), nest type (cavity/non-cavity) and coordinated parental care (uniparental/biparental or alloparental) were scored as binary, present or absent traits. High quality, close range images of adult birds were searched on photography sites online. Data on nesting behaviour were collected by searching online sources and life-history books. Data for parental care were obtained from Cockburn [11] (see the electronic supplementary materials for detail of trait classification). In total, 3544 species were sampled for iris colour, 1582 for nesting behaviour and 1326 species for parental care.

We used dated trees sampled from a full phylogeny of bird species [12] using the Hackett clade backbone [10]. Nodes in this tree are supported by a recently published phylogeny [13]. A maximum clade credibility (MCC) tree was determined for each set of 1000 trees using TreeAnnotator in BEAST v. 1.7 with posterior probability limit set at 0.5 [14]. An MCC tree is one in which the sum of scores for a clade (the posterior probability that the same clade is shared with the other trees) is highest. The final four trees were ultrametric, dated and fully resolved (i.e. no polytomies).

Ancestral traits of iris colour and cavity nesting were reconstructed based on maximum-likelihood using functions from the package phytools [15] in R [16]. Correlated evolution between cavity nesting and iris colour and parental care and iris colour were analysed using the DISCRETE module [17] in BayesTraits [18]. This tests whether the evolutionary model in which two traits evolved dependently on one another is significantly better than the evolutionary model in which two traits evolved independently. Significance for model comparisons was performed using likelihood ratio tests, with *α* set at 0.05. Likelihoods were estimated using 25 optimization attempts per run. All models were run with branches set to be equal as they had higher likelihoods than ultrametric trees.

## 3. Results

Ancestral reconstruction revealed that the ancestor of passerines was most likely a dark-eyed, cavity nesting bird (proportional likelihood 0.99 dark irides, 0.97 cavity nesting). The ancestor for passerines species that radiated following the split from the two extant basal wren species (*Acanthisitta chloris* and *Xenicus gilviventris*) was a non-cavity nester (proportional likelihood 0.88 non-cavity nesting). There were at least 275 independent transitions from dark eyes to bright eyes, and at least 39 independent transitions from non-cavity nesting to cavity nesting. A list of taxa with high instances of bright eyes and cavity nesting is provided in the electronic supplementary material.

**Figure 1.**
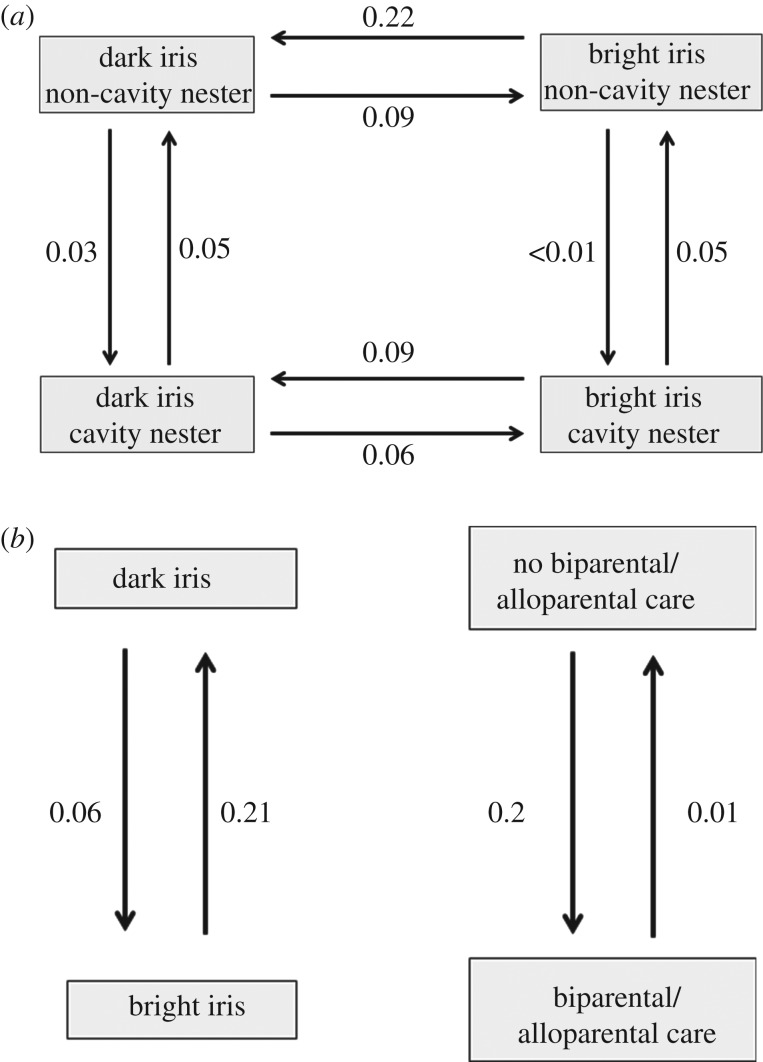
The most likely evolutionary models. (*a*) Iris colour and nesting behaviour evolve in a dependent manner; therefore the traits evolved sequentially (i.e. only one trait changes at a time). Rates away from bright irides are higher for non-cavity nesting birds than cavity nesting birds. (*b*) Iris colour and systems of parental care are not correlated. Changes between bright eyes and dark eyes occur independently from these traits.

The evolutionary model in which nesting behaviour and iris colour evolved dependently was significantly more likely than the evolutionary model in which two traits evolved independently from one another (log-likelihood assuming rate variation for dependent model, −924.50 versus independent model, −932.13; *χ*^2^ = 15.3, d.f. = 4, *p* < 0.01). Models were significantly worse when transition rates were constrained to be equal between dark eyes and bright eyes (table 1). Therefore, transition rate coefficients (i.e. the probability of changing from dark to bright and bright to dark) differ within trait environments (i.e. nesting). Rates of change away from bright eyes are higher in non-cavity nesting birds than cavity nesting birds. Rates of change from cavity nesting to non-cavity nesting are equal, regardless of iris colour (table 1 and figure 1*a*).

**Table 1.** Restricted models where rates of change between traits are constrained to be equal in their respective trait environments. Only changes from cavity to non-cavity nesters are equal regardless of iris colour.

trait change with rate restriction	log-likelihood (restricted)	log-likelihood (non-restricted)	*χ*^2^	*p*-value
dark to bright eyes in cavity nesters = dark to bright in non-cavity nesters	−926.60	−924.50	4.20	0.04
bright to dark eyes in cavity nesters = bright to dark eyes in non-cavity nesters	−927.39	−924.50	5.78	0.02
cavity to non-cavity nesters with bright eyes = cavity to non-cavity nesters with dark eyes	−924.55	−924.50	0.10	0.75
non-cavity to cavity nesters with bright eyes = non-cavity to cavity nesters with dark eyes	−927.27	−924.50	5.54	0.02

The evolutionary model in which iris colour evolved dependently with parental care was somewhat supported compared with the model in which the traits evolved independently, but this difference was not statistically significant (dependent log-likelihood = −792.35; independent log-likelihood = −797.53; *χ*^2^ = 8.36, d.f. = 4, *p* = 0.08; figure 1*b*).

## 4. Discussion

Our results indicate that iris colour evolution in birds may have occurred in a correlated manner with respect to nesting behaviour, but not parental care. Contrary to our first prediction, evolutionary transitions in iris coloration were unrelated to parental care. Moreover, contrary to our second prediction, cavity nesters are not under selection to evolve bright eyes. Instead our results show that non-cavity nesting birds are under strong selection to evolve dark eyes.

Predation pressure may be an important variable constraining the evolution of bright eyes in birds, as coloured eyes may be a conspicuous indicator of nest location. Bright irides in non-cavity nesters may be maladaptive because these birds would not have the anti-predator benefits of a concealed cavity (e.g. [19]). Alfred Russel Wallace [20] proposed that nesting behaviour would be a strong selective force in the evolution of dichromatic plumage in birds and would favour cryptic coloration in females incubating in open nests. In support of this hypothesis, Martin and Badyaev [21] found female plumage conspicuousness to be inversely related to nest predation, and Soler and Moreno [22] found conspicuous plumage more likely to evolve in cavity nesters than open nesters, albeit only in males. The need for visual concealment from predators may impose similar selection pressures on iris coloration, favouring transitions from bright to dark irides in non-cavity nesters.

A secondary trait related to cavity nesting, but not accounted for in this study, may be necessary for cavity nesters to evolve bright eyes. If bright eyes are important for guarding nests from competitors, we should expect selection for bright eyes in cases when competition over nest sites is particularly strong, for example if cavity availability is limited and/or neighbouring competitors are in close proximity (e.g. jackdaws [8]). Moreover, colour traits in plumage may also serve to appear conspicuous to nest competitors instead of bright eyes. Indeed, female *Eclectus* parrots (*Eclectus roratus*) have evolved conspicuous plumage in response to competition for nest cavities [23].

Contrary to the prediction that bright eyes may be beneficial for cooperative communication during reproductive efforts, the results found here suggest that biparental and alloparental care do not select for bright iris colour in passerine birds. It is possible that other measures of parental care such as long-term monogamy may be better predictors of reproductive synchronization. It has been proposed that birds that maintain their partnership across multiple breeding seasons may coordinate their actions through eye gaze [9], but whether or not synchrony between individuals is facilitated through gaze following requires empirical evidence. An alternative form of communication may involve eye flashing, where pupils rapidly dilate and contract independent of light condition (e.g. parrots [24]).

Animal pigmentation is frequently associated with sexual selection [3], though sexually dimorphic eye coloration is rare in passerines. To evaluate the potential role of sexual selection, it will be necessary to determine the extent to which individual eye colour varies (e.g. through spectral reflectance data), and if this variation may serve as an indicator of individual quality or as a result of arbitrary mate choice. Iris colour may also indicate sexual maturity or facilitate age recognition given that some bright-eyed adults have darker eyes as juveniles (e.g. [25]). This, however, does not explain interspecific variation in adult eye coloration.

The analyses performed here treated iris colour as a binary trait, but the evolution of a given colour may have different selection pressures. Salience of the eyes may be dependent on background colour such as plumage and environment (e.g. [23]). Moreover, non-passerines vary in the traits described here, and selection pressures may also be acting on their iris colour. We have demonstrated one possible mechanism by which selection could act on iris colour, and encourage further exploration into how this colour trait is related to competition, predation and sexual selection.

## Supplementary Material

Supplementary information

## Data accessibility

Data can be accessed at http://dx.doi.org/10.5061/dryad.ks824 [26].
