# Supplementary material for: Evolution of iris colour in relation to cavity nesting and parental care in passerine birds
Source: Biol Lett. 2017 Jan;13(1):20160783. doi: 10.1098/rsbl.2016.0783 (PMC5310583; doi:10.1098/rsbl.2016.0783)
Supplement: Supplementary information [file rsbl20160783supp1.docx]

Evolution of iris colour in relation to cavity nesting and parental care in passerine birds

Gabrielle L. Davidson, Alex Thornton, Nicola S. Clayton

**Supplementary Materials**

**Methods: Trait categorisation**

We defined birds to have coloured or ‘bright’ irises if the irides had pigmentation other than very dark brown or black (Fig S1). These colours include light brown, grey, red, yellow, blue, green and near white. High quality, close range images of birds were searched on photography sites online, and where possible, two or more photographs were used to confirm iris colour. Only adult birds were scored because changes in iris pigmentation from juveniles to adults is common in many passerine species (e.g. [1] and references therein). Species that were sexually dimorphic in iris colour such that one sex had dark brown or black eyes, and the other sex had bright eyes were excluded from the analysis to remove any effects of sexual selection on trait evolution (N=28 species out of a total of 3572 = 0.78%). We found two passerine species in our dataset to be polymorphic for iris colour independent of sex and included them in our analysis as bright-eyed: bearded tits, (*Panurus biarmicus*) [2] and Eurasian jays (*Garrulus glandarius*). Because dark eye coloration in bearded tits has only been reported at low frequencies in a single population (12-18%: Leighton Moss, UK) and was absent in a more recent report [1], we classified this species as having bright eyes. Eurasian jays had either blue or light brown eyes. Analyses run with birds with less conspicuous, light brown eyes (including the Eurasian jay) as being scored as being dark-eyed generated similar results if they were scored as bright eyed.


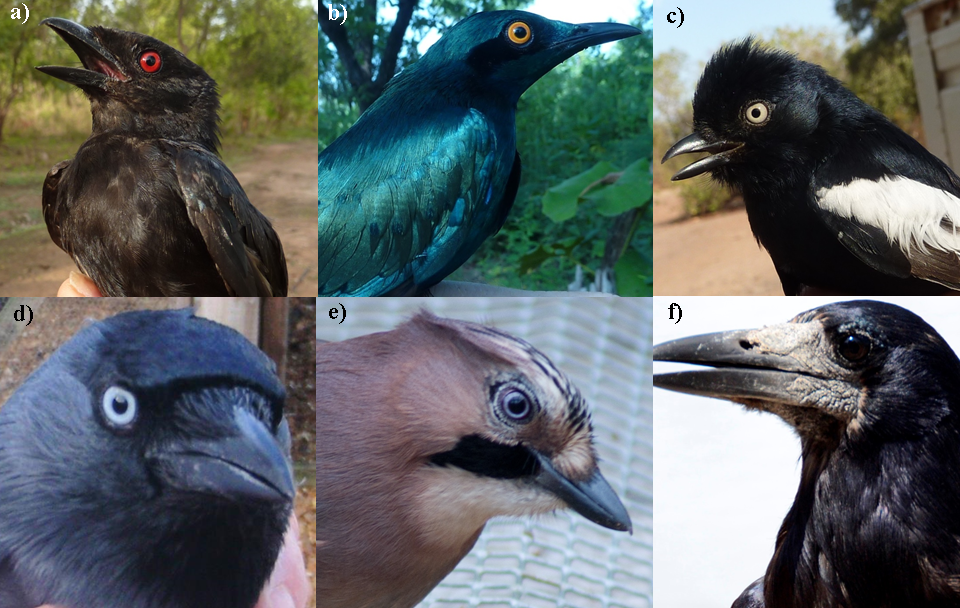


Figure. S1. Birds with bright (a-e) and dark (f) iris colour. a) *Dicrurus adsimilis;* b) *Lamprotornis chloropterus;* c) *Parus guineensis*; d) *Corvus monedula*; e) *Garrulus glandarius*; f) *Corvus frugilegus*. Photographs with permission from Tony Fulford (a-c), G Davidson (d), Julia Leijola (e), and Michael Lukas (f).

We defined birds to be cavity nesting if they built their nests in a sheltered chamber such as holes in trees, the ground, rock crevices, gaps in buildings or other such cavity, including both primary cavity nesters (those that excavate their own cavities) and secondary cavity nesters (those that rely on existing cavities). Birds were considered non-cavity nesters if they built an open cup or dome of nest material that did not reside within an existing chamber. Obligate pirate nesters (birds that steal other birds’ constructed nests) were excluded because nest type could vary, and brood parasites were excluded in the analyses performed with nesting type as they only use host nests to lay eggs. Data on nesting behaviour was collected by searching several sources online (e.g. Cornell lab of Ornithology, The Birds of North America Online, Handbook of the Birds of the World Alive, Bird Life Australia, CavNet, Cavity-Nesting Birds Research), in bird life history of books (e.g. [3]), and in published journals by searching the word “cavity nesting” in Google Scholar and ISI Web of Knowledge.

We classified parental care into two categories: care in which coordinated actions such as nest building, chick provisioning and predator scanning were expected between parents and/or helpers (1), and care in which coordinated actions were not expected (0). Coordinated care included species with biparental care and species with cooperative breeding. Cockburn [4] defined a species to have cooperative breeding if 10% or more of the nests within a population had more than two individuals assisting with reproductive efforts. No coordinated care included species where only the male or only the female provided care, and in species where no parental care was given (e.g. brood parasites). 1326 species (1048 biparental, 168 cooperative, 98 uniparental, 12 no parental) were analysed for correlated evolution between parental care and iris colour.

16% of species for iris colour (3554 species) and 10% of species for nesting traits (1582 species) were scored independently by a second observer (Cohen’s Kappa; iris colour = 0.75, nesting = 0.86). Any discrepancies were revisited to confirm the correct trait from multiple sources. If a consensus could not be achieved, that species was removed from the analysis.

**Overview of trait distribution and ancestral reconstruction**

Among the 3544 species sampled for iris colour, 786 species had bright eyes (22%). Of the 1582 species sampled for nesting behaviour, 557 species were cavity nesters (35%). Of the 1326 species included in parental care, 1216 had biparental or cooperative care (92%). The models in which transition rates between bright-eyed and dark-eyed birds, and cavity nesting and non-cavity nesting birds (i.e. from 0 to 1 and from 1 to 0) were allowed to vary across the tree was significantly better than the model in which they were constrained to be equal both for iris colour evolution (χ^2^=150.2, df=1, p < 0.001) and nesting evolution (χ^2^=12.4, df=1, p < 0.001). Therefore transition rate coefficients (i.e. the probability of changing from 0 to 1 and 1 to 0) differ within traits.

**Table S1. Iris colour distribution and ancestral states within clades**

| **Taxa** | **N** | **Bright eyes** | **Proportional likelihood (0)** | **Proportional likelihood (1)** |
| --- | --- | --- | --- | --- |
| Passeridae, Falconidae, Psittacidae | 3544 | 22% | 0.96 | 0.04 |
| Passeriformes | 3450 | 22% | 0.99 | 0.01 |
| Falconidae | 35 | 26% | 0.93 | 0.07 |
| Psittacidae | 59 | 39% | 0.99 | 0.01 |
| Thamnophilidae (antbirds) | 35 | 37% | 0.67 | 0.33 |
| Campephagidae (cuckooshrikes) | 13 | 46% | 0.25 | 0.75 |
| Platysteiridae (wattle-eyes, batises);  Malaconotidae (bush-shrikes) | 66 | 59% | 0.49 | 0.51 |
| *Oriolous* genus; Oriolidae (orioles) | 21 | 38% | 0.08 | 0.92 |
| Corvidae (crows, jays and allies) | 107 | 30% | 0.98 | 0.02 |
| Dicruridae (drongos) | 17 | 53% | 0.94 | 0.06 |
| Vireonidae (vireos, greenlets) | 53 | 38% | 0.99 | 0.01 |
| *Pychonotus* genus (bulbuls); Pycnonotidae | 39 | 36% | 0.50 | 0.50 |
| Leiothrichidae (laughing thrushes and babblers) | 92 | 50% | 0.90 | 0.10 |
| *Sylvia* (warbler) genus; Sylviidae | 24 | 75% | 0.91 | 0.09 |
| Genera including Prinius*,* Orthotomus and Apalis; Cisticolidae (Cisticolas) | 33 | 97% | 0.35 | 0.65 |
| Sturnidae (starlings) and Mimidae (mocking birds, thrashers) | 125 | 74% | 0.71 | 0.29 |
| some genera of Turdidae (thurshes) | 47 | 32% | 0.99 | 0.01 |
| *Ploceus* genus; Ploceidae (weavers) | 43 | 63% | 0.87 | 0.13 |
| *Cacicus* genus; Icteridae (Blackbirds) | 12 | 83% | 0.97 | 0.03 |
| *Ephagus* and *Quiscalus* genera (grackles); Icteridae | 7 | 86% | 0.08 | 0.92 |

**Table S1. Cavity nesting distribution and ancestral states within clades**

| **Taxa** | **n** | **Cavity nesters** | **Proportional likelihood (0)** | **Proportional likelihood (1)** |
| --- | --- | --- | --- | --- |
| Passeridae, Falconidae, Psittacidae | 1582 | 35% | 0.01 | 0.99 |
| Passeriformes | 1464 | 31% | 0.03 | 0.97 |
| Passeriformes (excluding *Acanthisitta chloris* and *Xenicus gilviventris* | 1462 | 31% | 0.88 | 0.12 |
| Falconidae | 26 | 73% | 0.00 | 1.00 |
| Psittacidae | 92 | 96% | 0.00 | 1.00 |
| Acanthisittidae (new zealand wrens) | 2 | 100% | 0.00 | 1.00 |
| Myiodynastes and Myiarchus genera; Tyrannidae (tyrant flycatchers) | 10 | 100% | 0.02 | 0.98 |
| Rhinocryptidae (tapaculos), Furnariidae (ovenbirds) | 107 | 90% | 0.03 | 0.97 |
| Climacteridae (australasian treecreepers) | 7 | 100% | 0.03 | 0.97 |
| Paridae (tits, chickadees) | 26 | 100% | 0.03 | 0.97 |
| Hirundinidae (swallows and martins) | 50 | 75% | 0.02 | 0.98 |
| Sturnidae | 96 | 86% | 0.00 | 1.00 |
| Muscicapidae (old world flycatchers) | 79 | 70% | 0.00 | 1.00 |
| Sittidae (nuthatches), Tichodromidae (wallcreepers), Certhiidae (treecreepers) | 57 | 56% | 0.00 | 1.00 |
| The *Sicalis* genus of the Thraupidae (tanagers) | 9 | 88% | 0.50 | 0.50 |
| The *Passer* and *Petronia* genera; Passeridae (old world sparrows) | 12 | 75% | 0.00 | 1.00 |

**Table S1 and S2.** Trait distribution and ancestral states within clades. S1 includes taxa with high instances of bright eyes (i.e. over 30%), (S2) includes taxa with high instances of cavity nesters (i.e. over 50%). Both tables include all passerines and outgroups. Proportional likelihoods represent the marginal likelihoods generated from rerootingMethod() in the “phytools” package for R (5,6). Models assume branch lengths are equal and that rates can vary across the tree. Values are given for the likelihood that the most recent common ancestor of each clade had S1) dark eyes (0) or bright eyes (1); S2) was not a cavity nester (0) or was a cavity nester (1).

References

1. Wilson,J. and Hartley,I.R. (2007). Changes in eye colour of juvenile bearded tits (*Panurus biarmicus*) and its use in determining breeding productivity. Ibis 149, 407-411.

2. Pearson, D.J. (1966) Observations on the iris colour of the Bearded Tit. Bird Study 13: 328-330

3. Snow DW, Perrins C. The birds of the Western Palearctic. Oxford: Oxford University Press; 1977.

4. Cockburn A. (2006). Prevalence of different modes of parental care in birds. Proc. R Soc. B. 273:1375.

5. R Development Core Team. (2011). A language and environment for statistical computing. (Vienna: R Foundation for Statistical Computing).

6. Revell L.J. (2012). phytools: an R package for phylogenetic comparative biology (and other things). Method Ecol. Evol. 3:217-223.

**Resource list for trait data**

**Sources for iris colour**

Roger Ahlman's Photo Galleries http://www.pbase.com/ahlman

James Lowen's Photo Galleries http://www.pbase.com/james_lowen

Peter Zwitser Wildlife and Nature Photography http://www.pbase.com/peterzwitser

Peter Ericsson's Photo Galleries http://www.pbase.com/peterericsson

Peter Bray Photo Galleries http://pbase.com/peterbray

Ingo Waschkie's Photo Galleries http://www.pbase.com/ingotkfr

Wong Tsu Shi's Photo Galleries http://www.pbase.com/wongtsushi

Margaret Sloan's Photo Galleries http://www.pbase.com/laumakani

Dubi Shapiro's Photo Galleries http://www.pbase.com/dubisha

Glenn Bartley Nature Photography http://www.glennbartley.com/

Robert Royse's North American Bird Photography http://www.roysephotos.com/BirdPhotoIndex.html

Antpitta: A Photo Gallery of Neotropical Birds http://antpitta.com/photos.htm

Birdway http://birdway.com.au/index.htm

BirdPhotos.com http://www.birdphotos.com/photos/index.php

Tanzanian Birds & Butterflies http://www.tanzaniabirds.net/

Arkive: Images of Life on Earth http://www.arkive.org/birds/

Oriental Bird Club Image Database http://orientalbirdimages.org/

The internet Bird Collection http://ibc.lynxeds.com

**Sources for nesting behaviour**

Beauty of Birds http://www.avianweb.com

Bird Forum http://www.birdforum.net

Cavity Nesting Bird Research http://www.cavitynester.org

Birds of North America Online (Cornell Lab of Ornithology) http://bna.birds.cornell.edu/bna/

Bradshaw, C.G. and Kirwan, G.M. (2008). A description of the nest of fiery capped manakin. Contiga. 4: 30-31.

Cockle, K.; Maders, C.; Di Santo, G.; Bodrati, A. 2008. The Black-capped Piprites Piprites pileata builds a spherical moss nest. Cotinga: 166-168

Cramp, S. et al. (1977-1996). Handbook of the birds of Europe, the Middle East and North Africa: The birds of the Western Palearctic. Vol 1- 9. Oxford University Press. Oxford

del Hoyo at al. (2003-2011). Handbook of the birds of the world, Volumes 8 - 16. Lynx Edicions, Barcelona.

deSanto, T.l., Wilson, M.F., Sieving, K.W., Armesto, J.J. (2002). Nesting biology of tapaculos (Rhinocryptidae) in fragmented south-temperate rainforests of chile. The Condor 104(3): 482-495.

Eggers, S., Griesser, M., Ekman, J.(2006) Predation risk induces changes in nest-site selection and clutch size in the Siberian Jay. Proceedings of the Royal Society B. 273: 701-706.

Greeney, H.F. (2009) Nest of the marble-faced bristle tyrant (Pogonotriccus ophthalmicus) with comparative comments on nests of related genera. J. of Ornithology. 121: 631-634.

Greeney, H.F., Sanchez, C., Sanchez, J.E., Carman, E. (2013) Nest and egg descriptions for genus Myrmeciza with the first description of nests and eggs of the dull-mantled antbird (M. laemosticta). J. Ornithology. 154: 1049 - 1056.

Handbook of the Birds of the World Alive http://www.hbw.com/

Hilty, S.L. (2002). Birds of Venezuela. Princeton University Press, Princeton, NJ.

Holden, P., Cleeves, T. (2006) RSPB Handbook of British Birds. A&C Black Publishers Lrd. London, UK.

Jing, Y., Fang, Y., Strickland, D., Lu, N., Sun, Y. (2009) Alloparenting in the rare Sichuan Jay (Perisoreus internigrans). The Condor. 111(4):662-667

Juniper, T., Parr, M. 1998. A guide to parrots of the world. Christopher Hlem Publishers, London, UK.

Kryukov, A., Iwasa, M.A., Kakizawa, R., Suzuki, H., Pinsker, W., Haring, E. (2004) Sycnhronic east-west divergence in azure-winged magpies (Cyanopica cyanus) and magpies (Pica pica).Journal of Zoological Systmatics and Evolutiory Research. 42(4)342-351.

McMullan, M., Donega, T.M., Quevedo, A. (2010). A field guide to the birds of Columbia. Fundacion ProAves, Columbia

Ming, M.A. (2011) Status of the Xinjiang Ground Jay: population, breeding ecology and conservation. Chinese Birds. 2(1): 59-62

Orians, G.H. (1983) Notes on the Behaviour of the Melodious Blackbird. The Condor. 85(4):453-460.

Pierce, A.J. 2007. Cooperative breeding in the puff-throated bulbul (Alophoixus pallidus) in Thailand. The Raffles Bulletin of Zoology. 55(1): 187-189.

Radnezhad, H., Satei, N., Kaboli, M., Karami, M., Khorasani, N., Prodon, R., Abari, M.F., Cheraghi, S. (2011)Breeding ecology of the Iranian ground jay (Podoces pleskei). African Journal of Biotechnology. 10(21):4494-4500.

Robinet, O.L. (1997). Ecology and conservation of the ouvea parakeet. PhD Thesis.

Rowley, I. (1972) CSIRO Wildlife Research. The Comparative Ecology of Australian Corvids IV. Nesting and the Rearing of Young to Independence. CSIRO Wildlife Research. 18(1): 91-129.

Salgado-Ortiz, J., Figueroa-Esquivel, E., Larios-Guzmaan, S., Robertson, R.J. (2001) Descriptions of Nests and Eggs of the Green-backed Sparrow and the Grey-throated Chat from the Yucatan Peninsula, Mexico. The Wilson Bulletin. 113(3):328-331.

Scheinder, N. A., Low, M., Arlt, D., Part, T. (2012) Contrast in edge vegetation structure modifies the predation risk of tural ground nests in an agricultural landscape. Plos ONE. 7(2): e31517

Schulenberg, T.S. (2010) Birds of Peru. Revised and Updated. Princeton University Press, Princeton, NJ.

Tozer, D.C. (2008). Nests of black-throated green warblers in tree cavities. The Wilson Journal of Ornithology 120(2): 409-412.

Van Balen, S., Eaton, J.A., Rheindt, F.E. (2011) Biology, taxonomy and conservation status of the Short-tailed Green Magpie from Java. Bird Conservation Intertional. 23(1): 91-109.

Warakai, D., Okena, D.S., Igag, P., Opiang, M., Mack, A.D. (2013). Tree cavity-using wildlife and the potential of artificial nest boxes for wildlife management in New Guinea. Tropical Conservation Science. (6):711-733.
